# Machine Learning-Based Characterization of *Bacillus anthracis* Phenotypes from pXO1 Plasmid Proteins

**DOI:** 10.3390/pathogens14101019

**Published:** 2025-10-08

**Authors:** William Harrigan, Thi Hai Au La, Prashant Dahal, Mahdi Belcaid, Michael H. Norris

**Affiliations:** 1 Department of Information and Computer Science, University of Hawai’i at Mānoa, Honolulu, HI 96822, USA; wlh3@hawaii.edu; 2 Pathogen Analysis and Translational Health Group, School of Life Sciences, University of Hawai’i at Mānoa, Honolulu, HI 96822, USA; thihaiau@hawaii.edu (T.H.A.L.); pdahal@hawaii.edu (P.D.)

**Keywords:** machine learning, *Bacillus anthracis*, anthrax, AI, phylogeny, affinity analysis, genotype to phenotype

## Abstract

The *Bacillus anthracis* pXO1 plasmid, encoding ~143 proteins, presents a compact model for exploring protein function and evolutionary patterns using protein language models. Due to the organism’s slow evolutionary rate, its limited amino acid variation enhances detection of physiologically relevant patterns in plasmid protein composition. In this study, we applied embedding-based analyses and machine learning methods to characterize pXO1 protein modules across diverse *B. anthracis* lineages. We generated protein sequence embeddings, constructed phylogenies, and compared plasmid content with whole genome variation. While whole genome and plasmid-based phylogenies diverge, the composition of proteins encoded along the pXO1 plasmid revealed lineage specific structure. Association rule mining combined with decision tree classification produced plasmid-encoded targets for assessing anthrax sublineage, which yielded functionally redundant protein modules that reflected geographic and phylogenetic patterns. A conserved DNA replication module exhibited both shared and *B. anthracis* lineage specific features. These results show that pXO1 plasmid protein modules contain biologically meaningful and evolutionarily informative signatures, exemplifying their value in phylogeographic characterizations of bacterial pathogens. This framework can be extended to study additional virulence plasmids across *Bacillus* and other environmental pathogens using scalable protein language model tools.

## 1. Introduction

Bacterial infectious diseases are a major component of morbidity and mortality globally. In 2019, one in eight global deaths were the result of bacterial infections, second only to ischemic heart disease [1]. This includes zoonoses that transfer from animals to humans and environmentally persistent pathogens. More advanced tools are needed to understand adaptations resulting in emergence of novel pathogens and antimicrobial resistance. These threats are the result of bacterial adaptation to diverse environments over time. Adaptations in functions such as infectivity or environmental persistence arise from underlying molecular sequences that are evolutionarily and functionally interdependent [2]. Mutations supporting pathogen function are maintained in bacterial populations due to evolutionary pressure from environmental stimuli, resulting in bacterial survival under adverse circumstances [3]. While contemporary methods (e.g., single nucleotide polymorphisms) effectively predict sites in individual genes that can be linked to functional adaptations, identifying co-occurring mutations across groups of genes or gene modules that are changing together over time under selective pressure is a challenge. Therefore, defining gene modules is a critical step in determining mutations linked to functional adaptations or phenotypes in bacterial species. To address this challenge, our study establishes a novel computational framework using protein language models to identify evolutionarily and functionally linked gene modules in a genetically homogenous pathogen.

*Bacillus anthracis* is a bacterial pathogen with a zoonotic reservoir and environmental persistence that we propose as the model organism to develop a framework for identifying gene modules that are evolutionarily and functionally linked. Anthrax can clinically present in diverse ways such as cutaneous, gastrointestinal, or inhalational, with inhalational being the rarest and most severe, and cutaneous being the most common and least severe. The global distribution of anthrax was recently modeled, identifying 63.5 million predominantly disadvantaged, rural livestock handlers at risk for disease [4]. Phylogenetically, *B. anthracis* is divided into 4 clades. Clade A *B. anthracis* has spread to all continents (but Antarctica) while B, C, and D are more isolated. *Bacillus anthracis* is a genetically homogeneous pathogen that slowly mutates with the highest level of genetic diversity present in southern Africa [5]. Due to long term quiescence in the soil, *B. anthracis* evolves slowly in comparison to bacteria that do not have a sporulation phase. This slower evolution has a dilative effect on adaptive mutations in the species, where we can observe mutations contributing to diversity and virulence under lower rates of accumulation.

Mutations maintained under positive selection often occur within functional modules, groups of genes or proteins that work together to perform specific biological functions. Gene expression and network analyses have shown that adaptive mutations can cluster within these modules, facilitating coordinated evolutionary changes [6,7,8,9]. Due to the slow evolution and high genetic homogeneity [5,10], *B. anthracis* provides a model organism to identify co-occurring proteins and predict functional modules where coincident adaptive mutations are likely to occur.

The pathogenicity of *B. anthracis* is dependent on two virulence-associated plasmids. The pXO1 plasmid encodes for the anthrax toxin components responsible for anthrax intoxication while the pXO2 plasmid proteins synthesize the protective capsule. Manifestation of anthrax disease depends on the presence of these two plasmids. Therefore, patterns of protein co-evolution on the pXO1 plasmid can provide insight into anthrax pathogenicity and plasmid maintenance by classifying past natural histories and identifying features linked to future functional trajectories.

Complex patterns in multi-allele protein co-occurrence can be difficult to assess. New computational technologies provide powerful functional prediction and pattern recognition capabilities. Machine learning approaches, particularly protein language models (pLMs), have emerged as powerful tools for predicting protein function from sequence alone [11]. By leveraging the “grammar” of amino acid sequences, pLMs generate representations that encode structural and functional properties, improving the annotation of uncharacterized proteins and improving the resolution of downstream protein co-occurrence predictions in genomic datasets. Recent applications in microbiology underscore their utility in pathogen genomics, where pLMs can reveal virulence factors, antimicrobial resistance determinants, and regulatory networks often overlooked by homology-based methods [12,13,14]. Combining pLM-derived representations with interpretable machine learning approaches, including association rule mining, manifold learning, and gradient-boosted trees, can systematically identify functional modules while maintaining biological interpretability.

In this study, we use the pXO1 plasmid of *B. anthracis* as a tractable model system to investigate protein co-occurrence and functional module identification with protein language models. We applied embedding-based analyses to characterize the ~143 proteins encoded on pXO1 across 73 phylogenetically diverse *B. anthracis* strains. Our approach combines sequence embeddings with association rule mining and phylogenetic analysis to identify co-evolving protein modules and assess their relationship to lineage-specific adaptations. By comparing plasmid-based protein variation patterns with whole genome phylogenies, we aimed to determine whether pXO1 encodes biologically meaningful evolutionary signatures that reflect the organism’s geographic distribution and pathoadaptive history. Functional modules of pXO1 important for plasmid maintenance, transfer, and pathogenicity were explored using pLM embeddings and machine learning. This framework provides a scalable approach for understanding virulence plasmid evolution that can be extended to other pathogens with similar plasmid-borne virulence dependencies.

## 2. Materials and Methods

### 2.1. Extraction of pXO1 Amino Acid Sequences and Embedding Generation

The final dataset consisted of 73 complete pXO1 plasmid sequences selected to capture broad phylogenetic diversity across *B. anthracis*. These sequences were sampled from the three major *B. anthracis* clades (A, B, and C) and encompassed 13 defined sublineages. Although clade D is considered a major clade of *B. anthracis* it represents a rare and potentially historical group of anthrax lineage [15], therefore this analysis focuses on clades A and B, which account for the majority of global anthrax cases, with clade C included for completeness despite its limited prevalence [5,16,17]. All protein-coding sequences associated with these plasmids were retrieved from the NCBI Genome database (accession numbers available in the project’s GitHub repository (https://github.com/Hawaii-Bioinformatics/B-anthracis-pXO1-analysis/: accessed on 28 August 2025), yielding a total of 2123 representative proteins.

To generate vector representations of the protein sequences, we employed a version of the ESM-2 protein language model with 36 layers and 3 billion (3B) parameters that was fine-tuned on viral proteins [11,18]. The 36-layer, 3B-parameter version of ESM-2 was selected because it balances representational capacity with computational efficiency. Larger models with more parameters (e.g., the 15 billion (15B)-parameter ESM-2) provide marginal gains in downstream biological tasks at substantially higher computational cost [11]. Unlike structure-based approaches such as AlphaFold2, which outputs atomic coordinates optimized for single-protein modeling, ESM-2 generates sequence-level embeddings that are well-suited for pairwise alignments and high-quality functional annotation [11]. To expand the coverage of accessory and host-interacting protein families, we chose a version of the ESM-2 3B-parameter model that had been fine-tuned on viral protein sequences. Fine-tuning on viral sequences enhanced the capability of the base ESM-2 model to generalize to sequences beyond the bacterial and plasmid sequences used in the initial pre-training [18]. The fine-tuned ESM-2 model used in this study yielded embeddings that capture the remote homology and diversity present in *B. anthracis* pXO1 genes without compromising performance on bacterial or plasmid sequences.

### 2.2. Functional Annotation of Protein Sequences

For sequences lacking functionally specific annotations in the downloaded NCBI dataset, we performed soft-alignments of ESM-2–derived protein embeddings against the UniRef50 database, following a previously published protocol [19]. These uncharacterized pXO1 proteins were assigned functional labels based on the top-scoring soft-alignment match. This embedding-based approach leverages semantic similarity in protein representation space, enabling more accurate annotation of previously ambiguous sequences than homology-based sequence similarity methods.

### 2.3. Phylogenetic Tree Construction and Visualization

**Figure 1:**
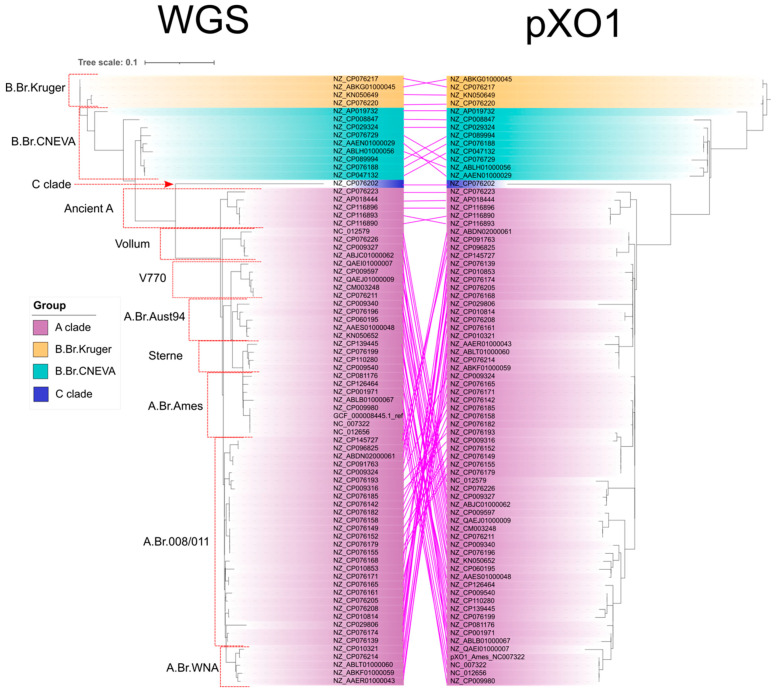
Phylogenetic trees built from whole-genome sequencing (WGS) and pXO1 plasmid sequencing of *Bacillus anthracis* strains. Branches represent individual anthrax strains. Branch highlights indicate strain lineage from three main *B. anthracis* ancestral lineages (A, B and C). Red borders and corresponding labels indicate placement and name of *B. anthracis* sublineages. Pink edges indicate strain placement across phylogenetic trees.

Phylogenetic trees of whole-genome and complete pXO1 plasmid sequences from 73 *B. anthracis* strains were generated to assess consistency between the phylogenies (Figure 1). Sequence data were retrieved from the NCBI genome database and analyzed using the PhAME pipeline as previously described [20,21,22]. All genomes and plasmids were processed on the University of Hawai‘i’s KOA high-performance computing cluster using PhAME’s default parameters for alignment and phylogenetic reconstruction [23].

The resulting Newick-format trees were visualized using the Interactive Tree of Life (iTOL) platform [24], and phylogenetic relationships were manually assessed to verify congruence with known lineage structure. Following assessment, two strains (NZ_CP076168, and NZ_CP089994) that were initially included in the WGS and pXO1 phylogenetic trees were excluded from downstream analyses due to evidence of lineage mis-annotation based on anomalous protein variation. GCF_000008445.1_ref was also excluded from downstream analyses as its purpose was to serve as a reference for verifying WGS tree topology.

### 2.4. Comparison of pXO1 Protein Composition to Whole-Genome Phylogeny

**Figure 2:**
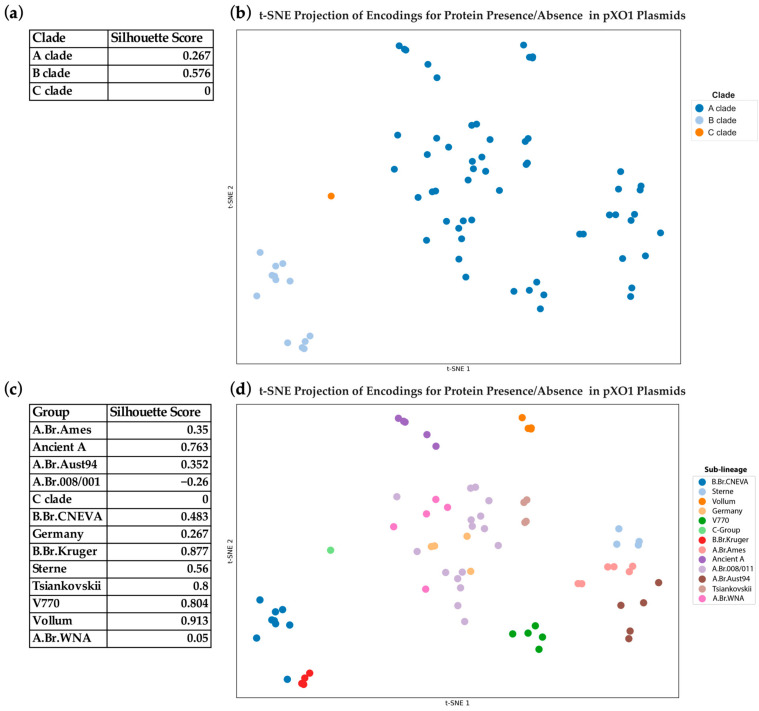
(**a**) Silhouette scores of three major *B. anthracis* ancestral clades (A, B and C). (**b**) The t-SNE projection of pXO1 plasmid representations colored according to clade (n = 73). (**c**) Silhouette scores of common *B. anthracis* sublineages. (**d**) T-distributed stochastic neighbor embedding (t-SNE) projection of pXO1 plasmid representations colored according to thirteen sublineages (n = 73).

To assess whether the protein composition of *B. anthracis* pXO1 plasmids reflects lineage, each pXO1 plasmid was converted to a 2123-element binary vector, with positions corresponding to each unique protein sequence variant present in the 73 pXO1 plasmid sequences, as defined by the NCBI genome database. At each position, a value of 1 was assigned when the plasmid contained the protein sequence variant; otherwise, the value was 0. These high-dimensional vectors were projected into two dimensions with t-distributed stochastic neighbor embedding (t-SNE) [25] (Figure 2). Clustering of the t-SNE projections was then compared with lineage and sublineage assignments from the whole-genome phylogeny to evaluate concordance between plasmid protein composition and chromosomal evolutionary structure.

### 2.5. Affinity Analysis of pXO1 Proteins

The genotypic traits underlying the pXO1 plasmid representation clustering patterns were explored using a combination of mutual information and affinity analysis. Mutual information was used to identify pXO1 plasmid proteins most informative of *B.anthracis* sublineage classification. Binary presence/absence vectors for plasmids (see Section 2.4) were used as input features, and known sublineage labels were used as the target variable. Mutual information scores were calculated using the mutual_info_score function from scikit-learn (v1.4.2). For each sublineage, mutual information was computed using a one-vs-all approach, in which the target variable was binarized to distinguish the focal sublineage from all others. Proteins with mutual information scores greater than 0.1 in any sublineage were considered informative and retained for subsequent affinity analysis.

The affinity analysis was performed using a market-basket algorithm, implemented via the mlxtend library (https://rasbt.github.io/mlxtend: accessed on 15 March 2025), on the proteins identified by mutual information. The market-basket algorithm identified protein sets with a support value above 0.1 and a confidence score exceeding 0.9. Sequences present in the protein sets were further utilized to develop a *B. anthracis* lineage classifier and define phylogenetically informative protein modules.

### 2.6. Bacillus anthracis Lineage Classifier

**Figure 3:**
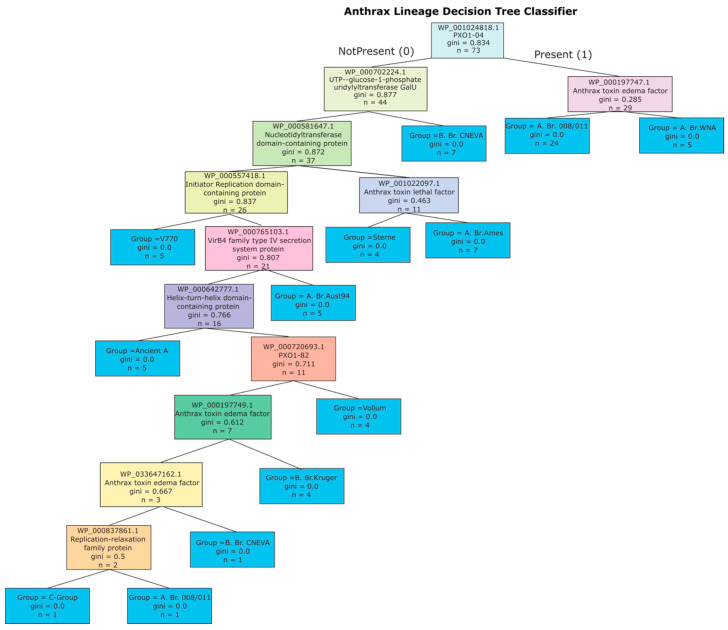
Decision tree-based classifier for allele occurrence in *B.anthracis* sublineages. Branches on left indicate protein absence (0) and right pointing branches indicate protein presence (1). Nodes (colored boxes) represent allelic variants labeled with the corresponding protein sequence identifier, protein function, gini score and number of strains (n). Leaves (blue boxes) represent sublineages labeled with gini score and number of strains.

Protein sequences identified in the affinity analysis were encoded as binary presence/absence features and fed into a Gini-impurity decision tree implemented with scikit-learn (v 1.4.2) [26]. Default hyper-parameters were used (criterion = “gini”, splitter = “best”, max_depth = None), and the resulting model classified the 73 strains into eleven *B. anthracis* sub-lineages using these proteins as molecular markers (Figure 3).

### 2.7. Protein Module Characterization

The functional annotations of sequences used in the *B. anthracis* lineage classifier were used to define protein modules. To construct these modules, protein sequences from the pXO1 plasmid dataset annotated with any function identified in the decision tree were retained. This filtering resulted in a final set of sequences corresponding to the functional annotations used in the classifier.

**Figure 4:**
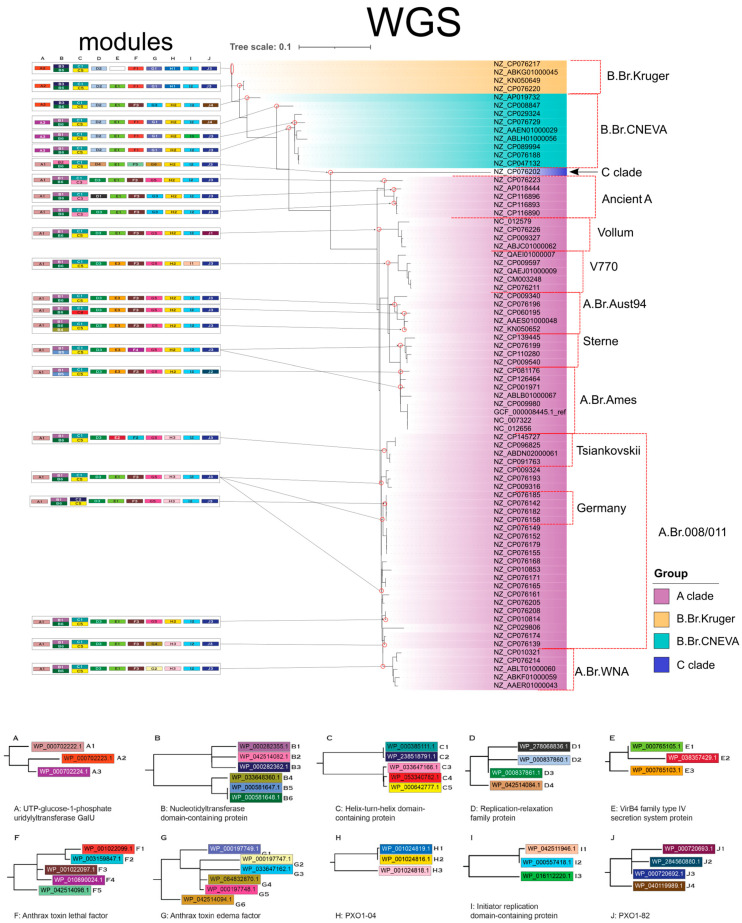
*Bacillus anthracis* pXO1 protein modules (left; 23 modules) mapped onto the whole-genome sequence phylogeny (right; 73 *B. anthracis* strains). WGS label colors indicate ancestral lineage. Red borders and corresponding labels indicate placement and name of *B. anthracis* sublineages. Mini-trees (bottom; **A**–**J**), labeled alphabetically, represent the ten protein functions used to define modules, with branches corresponding to alleles. Colors in the mini-trees match the proteins in each module. Red circles on phylogeny branches indicate the presence of protein modules in specific strains or corresponding sublineages.

**Figure 5:**
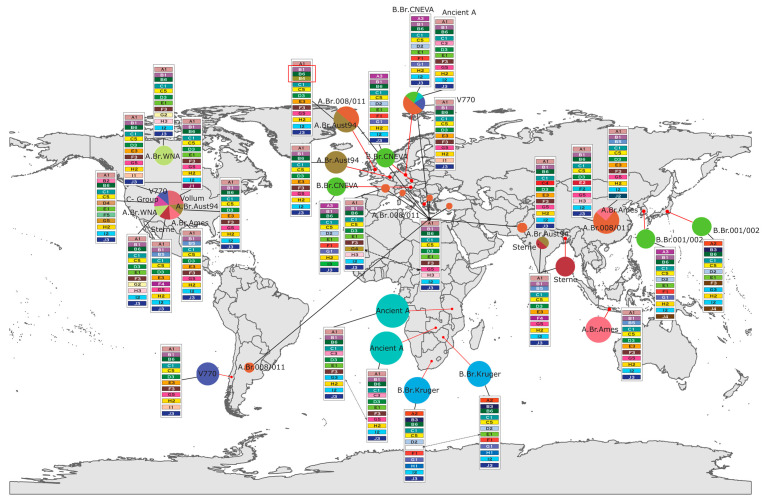
Global distribution of *B. anthracis* protein modules by sample collection location. Pie charts represent the proportion of strains from each sublineage sampled within a country (23 countries, 13 sublineages, 73 strains), with colors corresponding to sublineage classification. Rectangular blocks depict protein modules, with alphabetic labels for functions and allele colors consistent with mini-trees shown in Figure 4. Red lines map geographic locations to the pie-charts while black lines connect lineages in pie-charts to the protein module profiles. Red boxed protein modules indicate geographically unique module combinations. Dashed arrows between modules indicate specific protein allele differences in geographically localized lineages.

Each protein module consists of a distinct allelic variant for each annotated function, such that modules share the same protein functions but differ in their specific sequence composition. These modules were mapped onto the whole-genome phylogenetic tree (see Section 2.3) and a global geographic map to visualize their distribution across evolutionary lineages and sampling locations (Figure 4 and Figure 5). Protein similarity dendrograms (mini-trees) for proteins of the same functional annotation were generated using MAFFT [27,28].

### 2.8. Domain Analysis on Functionally Redundant Module Proteins

To investigate intra-plasmid protein redundancy, we searched the modules defined in Section 2.7 for proteins that shared a functional annotation yet occurred more than once on the same pXO1 plasmid. Eleven such cases were found—six nucleotidyltransferase domain-containing proteins and five helix–turn–helix (HTH) domain-containing proteins.

**Figure 6:**
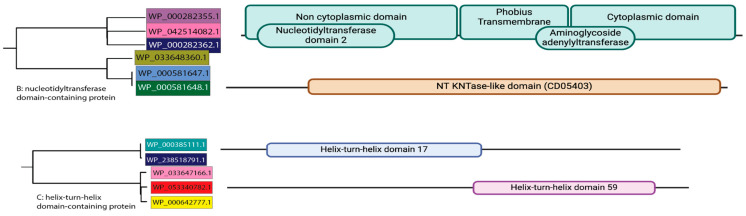
Protein similarity dendrogram (left) for intra-plasmid redundant protein functions within pXO1 protein modules (consistent with mini-trees in Figure 4B,C). Hypothetical protein sequence structure representing protein domains and superfamilies present in alleles according to InterProScan (right). Domain and superfamily placements along the hypothetical protein sequence reflect their positions as identified by InterProScan. Note: WP_000385111.1 and WP_238518791.1 are identical amino acid sequences under different sequence identifiers.

Domain architectures were characterized with InterProScan [29] (Figure 6). For each redundant protein, all InterPro domains were cataloged. The resulting domain profiles were used to evaluate why multiple copies of similarly annotated proteins coexist, considering possibilities such as domain diversification, functional sub-specialization, or regulatory redundancy.

### 2.9. DNA Processing and Replication Module

The nuclease-related domain (NERD) on the pXO1 plasmid has been shown to possess nuclease activity and to participate in DNA-processing functions [30]. In *B. anthracis* and related species, NERD often co-occurs with proteins that form DNA-replication or DNA-repair modules.

NERD has been detected in 10,756 bacterial taxa. STRINGDB’s gene-co-occurrence analysis yields an 11-gene NERD network, represented here by sequences from *Caldanaerobacter subterraneus*. From these 11 genes, we extracted 57 reference domains and superfamilies using STRINGDB annotations and InterPro [29,31]. We then scanned all proteins encoded by the 73 pXO1 plasmids with InterProScan, searching for homologues of any reference domain.

Proteins containing at least one reference domain were retained and grouped into a putative “DNA-processing module,” representing plasmid-encoded factors likely involved in replication, repair, or related functions.

**Figure 7:**
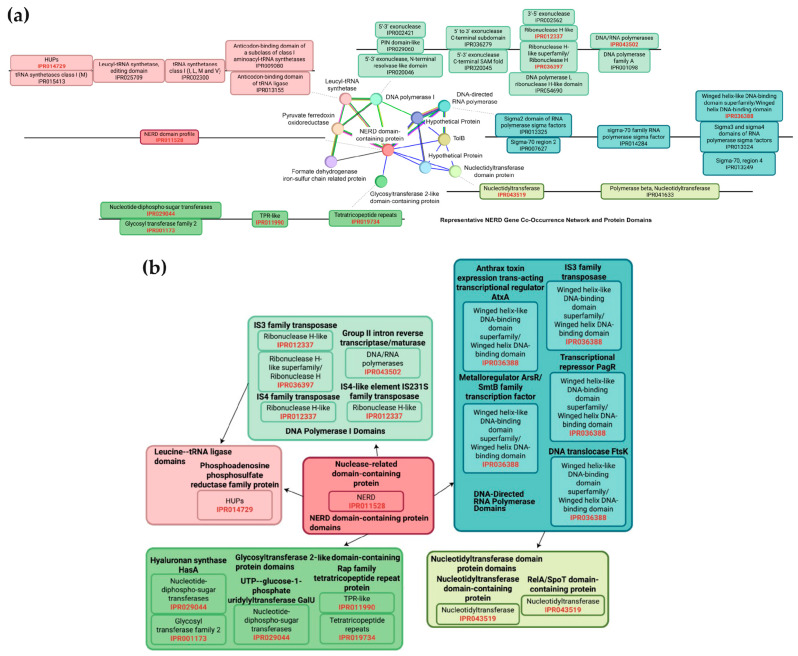
(**a**) NERD protein association network from STRINGDB with representation of protein sequences and their domains. Proteins in the network were found to co-occur with the NERD protein across bacterial species. (**b**) Theoretical representation of predicted DNA-processing related domains identified along the pXO1 plasmid. Colored and labeled according to proteins in the Representative NERD Gene Co-occurrence Network and Protein Domains. Identifiers colored red represent domains present in the reference DNA-processing network and the putative pXO1 DNA-processing module. Edges represent associations present in the STRINGDB reference network. Colors in NERD protein association networks map to colors in Table 1.

To deepen module discovery across *B. anthracis*, we anchored our search on the nuclease-related domain (NERD), chosen because it is enzymatically characterized on pXO1 [30], occurs in more than 10,000 bacterial taxa, and consistently co-occurs with replication- and repair-associated proteins [31]. We extracted the STRINGDB co-occurrence network for a canonical NERD protein from *Caldanaerobacter subterraneus*, cataloged the 57 distinct InterPro domains and superfamilies present in the network’s eleven genes, and scanned the 73 pXO1 proteomes with InterProScan; any protein harboring at least one reference domain was retained (Table 1 and Figure 7). These proteins constitute a putative DNA-processing module whose composition can now be compared across lineages to uncover *B. anthracis*–specific differences in plasmid architecture.

## 3. Results

### 3.1. Bacillus anthracis Whole Genome Sequencing and pXO1 Plasmid Sequencing Produce Inconsistent Phylogenies

Phylogenetic trees constructed from *B. anthracis* whole genome sequences and pXO1 plasmid sequences displayed notable topological inconsistencies (Figure 1). While the broad separation between the A and B clades remained consistent across both approaches, the placement of specific strains varied substantially, particularly within the A clade.

Strains belonging to the Ancient A sublineage were the most consistently positioned in both phylogenies; however, other strains of A sublineages, such as Vollum, Sterne, and A.Br.WNA, exhibited markedly different placements, undermining confidence in the congruency between the two sequencing strategies. Discrepancies in the phylogenetic placement of certain B clade strains were also observed, though to a lesser extent.

These results highlight the limitations of plasmid-based phylogenetic inference and suggest that plasmid and whole genome data may reflect distinct evolutionary histories. The observed inconsistencies underscore the need for phylogenomic approaches that yield reliable and coherent results, regardless of whether analyses are based on plasmid or whole genome data.

### 3.2. Vector Encodings of pXO1 Plasmid Protein Composition Reflect Lineage-Specific Structure

We utilized t-SNE to reduce the dimensionality of presence–absence vector encodings representing pXO1 plasmid protein composition to investigate lineage-specific clustering patterns (Figure 2). The plasmid representations clustered according to known sublineages, indicating that the composition of proteins at the plasmid-level reflects underlying phylogenetic structure captured by *B. anthracis* whole genome sequencing.

The overall mean silhouette score for the three major clades (A, B and C) was 0.422, while the mean silhouette score across the 13 defined sublineages was slightly higher at 0.458, suggesting finer-scale clustering resolution.

The *B. anthracis* B sublineages B.Br.Kruger and B.Br.CNEVA clustered closely together, while A clade genomes grouped more distantly from one another, with the C clade forming an intermediate bridge, mirroring patterns observed in the whole genome sequence phylogeny. The most compact and cohesive clusters corresponded to the Vollum (silhouette = 0.91), B.Br.Kruger (0.88), and V770 (0.80) sublineages, each of which is also geographically constrained.

In contrast, the A.Br.008/011 sublineage, the most abundant and geographically widespread lineage in the dataset, clustered near other boundaries (silhouette = –0.26). This pattern reflects its broad geographic distribution and overlap with multiple sublineages, and the high genetic homogeneity characteristic of *B. anthracis*. The species alternates between brief periods of rapid replication in hosts and long phases of dormancy as spores, limiting opportunities for diversification and preserving conserved genomic features [32]. As a result, dimensionality reduction in gene presence-absence vectors captures shared traits across lineages, contributing to the diffuse placement of A.Br.008/011 near related sublineages [5,33,34,35].

To further validate these patterns, we calculated additional clustering metrics. At the ancestral clade level (A, B, C), the adjusted Rand index (RI) was 0.680 and clustering accuracy was 0.712, consistent with broad but partially overlapping separation of the three major lineages. At the sublineage level, the RI increased to 0.914 and accuracy was 0.685, reflecting strong concordance with phylogenetic assignments.

These findings reinforce the observation that plasmid protein composition encodes lineage-specific signals and reflect broader patterns observed in whole-genome phylogenies.

### 3.3. Decision Tree of pXO1 Protein Composition Identifies Sublineage-Specific Markers

Association rule mining refined by mutual information analysis of pXO1 plasmid composition yielded a decision tree comprising ten unique protein functions and twelve distinct protein sequences informative of *B. anthracis* sub-lineage classification (Figure 3). This decision tree demonstrates how a minimal set of pXO1-encoded proteins can accurately discriminate among clades and sublineages. Each internal node represents the presence or absence of a protein variant that provides discriminatory power, and progressing down the tree recursively partitions the dataset until sublineages are resolved at the terminal nodes.

Several of the most informative splits are driven by canonical virulence determinants. For example, anthrax toxin edema factor (EF) sequences WP_000197747.1, WP_000197749.1, and WP_033647162.1 distinguish the A.Br.WNA, B.Br.Kruger, and B.Br.CNEVA modules, respectively. Similarly, the anthrax toxin lethal factor (LF) sequence WP_001022097.1 is uniquely associated with the A.Br.Ames lineage. These findings underscore the central role of toxin variants in differentiating sublineages, consistent with their importance in anthrax pathogenesis [36].

Functional studies provide strong support for these virulence determinants. Edema factor (EF) and lethal factor (LF) are highly conserved across all *B. anthracis* lineages, with >95% amino acid identity and retained catalytic activity, and both have been experimentally validated as essential components of anthrax toxin action. LF functions as a zinc-dependent metalloprotease targeting MAPKKs to induce immune cell death, whereas EF is a calmodulin-dependent adenylate cyclase responsible for edema through cAMP dysregulation [37,38,39,40]. While no lineage-defining EF or LF variants have been described, the classifier’s recovery of these canonical virulence markers reinforces that our approach captures biologically meaningful signals and highlights allelic diversity that may stratify sublineages.

Replication-associated proteins also emerged as key discriminators. The replication-relaxation family protein and the initiator replication domain-containing protein, both implicated in DNA binding during pXO1 replication [41,42,43,44], separate the sublineages. Specifically, the absence of replication-relaxation protein variant WP_000837861.1 characterizes the C-clade, while the absence of initiator replication protein allele WP_000557418.1 distinguishes the V770 sublineage.

Additional discriminatory power is provided by proteins involved in secretion and mobilization. The VirB4 family type IV secretion system protein (WP_000765103.1), which mediates translocation of virulence factors into host cells [45], is a specific marker of the A.Br.Aust94 sublineage. Proteins such as PXO1-04 and PXO1-82, though poorly characterized, consistently co-occur with proteins central to virulence and replication, suggesting they may be functionally important components of pXO1 biology.

### 3.4. Protein Modules Reflect Whole Genome Phylogenetic Structure

We used ten protein functions identified in the sublineage classifier (Figure 4) to define protein modules from the pXO1 plasmid that reflect the sublineage structure observed in the whole-genome phylogeny. This process resulted in 23 distinct protein modules that correspond to both ancestral lineage and sublineage patterns in the WGS phylogenetic tree. These modules follow phylogenetic topology, map to isolated groups, and highlight differences in allele occurrence across diverse *B. anthracis* strains (Figure 4).

The C-clade formed a distinct group in the phylogeny and was characterized by a unique protein module consisting of three non-redundant sequences corresponding to the replication-relaxation family protein, anthrax toxin lethal factor, and anthrax toxin edema factor. This module was the most compositionally distinct among all lineages, corroborating findings in both the WGS-tree and the t-SNE plasmid representation analysis, that the C-clade is the most distinct lineage, and appears to be more similar to both lineages A and B than A and B are to each other.

Within the protein modules, protein functions were identified to have strong clade specificity. For example, UTP-glucose-1-phosphate uridylyltransferase (GalU) showed three clade-restricted variants: WP_000702223.1 and WP_000702224.1 were exclusive to the B-clade, whereas WP_000702222.1 was found only in the A and C clade. Similarly, replication-relaxation family proteins demonstrate lineage specificity, with WP_00837860.1 exclusive to the B clade, WP_042514084.1 to the C clade, and WP_278068836.1 and WP_000837861.1 to the A clade.

At the sublineage level, we observed further differentiation using additional proteins. The PXO1-04 protein (WP_001024819.1) was specific to the B.Br.Kruger sublineage, and the V770 sublineage was the only group containing WP_042511946.1, a variant of the initiator replication domain-containing protein. The Tsiankovskii sublineage distinctly contains the WP_038357429.1 sequence of the VirB4 family type IV secretion system protein, and the A.Br.WNA sublineage was the only sublineage to contain the anthrax toxin edema factor sequence WP_000197747.1. The Sterne module was the only lineage to have the anthrax toxin lethal factor sequence WP_010890024.1.

The protein modules further expand on the findings of our decision tree analysis, identifying biologically important protein functions that have lineage and sublineage sequence specificity. These protein modules provide a consistent and informative framework relying only on the composition of the pXO1 plasmids and reflect the lineage and sublineage relationships observed in the WGS-based phylogenetic tree.

### 3.5. Geographic Distribution of Protein Modules Reveals Region-Specific Genomic Variation

The geographic distribution of *B. anthracis* protein modules provides context to allele occurrence patterns that reflect the phylogenetic structure. Spatial mapping of module composition using epidemiological metadata uncovered regional trends, indicating that specific genomic features are associated with distinct geographic areas (Figure 5). These patterns provide insight into clade- and sublineage-level diversification observed in the whole-genome phylogeny as *B. anthracis* spread around the globe.

Within the Ancient A sublineage, protein module variation was observed among strains sampled from Zambia, Tanzania, and Germany. The Zambian (n = 1) and German Ancient A strains (n = 1) contained the anthrax toxin edema factor allele WP_000197748.1, while Tanzanian strains (n = 3) harbored the WP_033647162.1 variant. All ancient A strains share the WP_000837861.1.1 allele of the replication-relaxation family protein and initiator replication domain-containing allele WP_000557418.1. These findings show that despite geographic proximity, African strains differ in toxin edema gene profiles but retain identical plasmid replication machinery.

The A clade, the most globally widespread and abundant lineage in the dataset, showed both geographic conservation and within-clade diversification in protein module composition. The anthrax toxin lethal factor allele WP_001022097.1 was conserved across A clade sublineages in Europe. However, divergence in this allele was observed in Sterne strains (South Asia, n = 3; North America, n = 1) and Tsiankovskii strains (China, n = 4), indicating potential regional adaptation in toxin-related genes.

The B clade also exhibited geographically influenced variation in module composition providing context to phylogenetic relationships captured from WGS. The B.Br.001/002 strain from Japan (n = 1) shared greater similarity in protein module composition with B.Br.Kruger strains from Southern Africa (n = 4), whereas the European B.Br.CNEVA strains (n = 8) more closely resembled a B.Br.001/002 strain from South Korea (n = 1). These relationships were most apparent in the identical alleles of the UTP–glucose-1-phosphate uridylyltransferase GalU and nucleotidyltransferase domain-containing proteins shared across regions. Despite their classification within the same sublineage, the distribution of these alleles suggests independent geographic origins and region-specific evolutionary trajectories.

Overall, the geographic distribution of protein modules revealed lineage-specific patterns and highlighted both conserved and divergent genomic features, providing context for the relationships observed in the whole-genome phylogeny.

### 3.6. Domain Analysis of Ambiguously Annotated Proteins Reveals Complementary Functional Roles

Across the pXO1 plasmids analyzed, multiple proteins annotated with the same functional annotation were observed to co-occur. In effect, each plasmid contained two to three nucleotidyltransferase domain–containing proteins and two to three helix–turn–helix (HTH) domain–containing proteins. This consistent co-occurrence of identical functional annotations within individual plasmids raises the question of whether nucleotidyltransferase- and HTH-domain containing proteins encode conserved non-redundant functions. The domains of these co-occurring proteins were further analyzed to understand their functional relationships.

This analysis revealed that the recurrence of the nucleotidyltransferase- and HTH-domain containing protein annotations is due to the presence of structurally distinct domains that, when co-occurring, likely participate in a coordinated biological function. Specifically, proteins annotated as nucleotidyltransferase domain–containing occurred two to three times per plasmid and included several distinct functional domains (Figure 6). Proteins containing a nucleotidyltransferase domain-2 and an aminoglycoside adenylyltransferase domain co-occurred with proteins that carried a kanamycin nucleotidyltransferase (KNTase)-like domain. Nucleotidyltransferase domain 2 and the aminoglycoside adenylyltransferase domain, both of which catalyze adenylylation (nucleotidylation) of aminoglycoside antibiotics, are known to inactivate antibiotics such as kanamycin, streptomycin, and spectinomycin [46,47,48]. The presence of the KNTase-like domain, a specialized variant targeting aminoglycosides with a 4′- or 4′′-hydroxyl group, may indicate substrate-specific resistance. The co-occurrence of these domains suggests a modular resistance mechanism, where overlapping but distinct enzymatic activities broaden the spectrum of aminoglycoside inactivation. While this may provide adaptive value for plasmid maintenance, particularly in soil environments where aminoglycosides are naturally produced by *Streptomyces* species, direct evidence of such selective pressure in *B. anthracis* is lacking. Thus, their recurrence on pXO1 is best interpreted as a potential functional feature, warranting further investigation rather than a demonstrated consequence of environmental antibiotic exposure.

Similarly, helix–turn–helix (HTH) domain-containing proteins were present two to three times per plasmid on average. We identified a total of three distinct HTH domain-containing protein combinations across the dataset. Previous characterizations of the HTH domain 17 (a DNA-binding motif) identified this domain to consistently associate with excisionase-like proteins [49,50]. This relationship is typically observed in DNA mobilization pathways [51] In this dataset of pXO1 plasmids, the HTH domain 17 co-occurs with the relatively uncharacterized HTH domain 59. The conserved co-occurrence of HTH domain types suggests potential functional modularity in DNA mobilization but requires further experimental validation to characterize the exact relationship.

These findings support that the repeated appearance of protein functions within pXO1 plasmids may exhibit modularity in which distinct yet complementary domains cooperate to perform broader biological functions, such as antibiotic resistance or DNA mobilization. This functional insight was not apparent from standard annotations, underscoring the value of domain-level characterization to resolve ambiguous protein functions and uncover latent protein–protein relationships.

### 3.7. Characterization of a DNA Replication Module Reveals Conserved and Lineage-Specific Protein Functions on the pXO1 Plasmid

Proteins involved in DNA processing and replication in bacteria are typically informative of various lifestyle and phylogenetic traits. Therefore, we defined a DNA processing module of pXO1 plasmid-encoded proteins using domain-based annotation derived from a reference nuclease related gene co-occurrence network (Figure 7a). The annotation of the DNA processing module was guided by the characterization of the nuclease-related domain (NERD) protein by Grynberg and Godzik [30], a known DNA-processing nuclease found on the pXO1 plasmid. We compiled a set of reference protein domains and superfamilies from a representative bacterial NERD-centered co-occurrence network of 11 proteins (Table 1). Reference domains and superfamilies were extracted from STRINGDB and InterPro and used to identify functional analogs within our dataset of the proteins that occurred more than once in the 73 pXO1 plasmids (Figure 7).

We identified 57 protein sequences representing 16 protein functions that contained at least one domain shared with the reference network (Figure 7b). All 16 protein functions were conserved in every pXO1 plasmid analyzed. The reference domains and superfamilies found in the pXO1 plasmids are from six protein groups from the representative network: DNA polymerase I, DNA-directed RNA polymerase, nucleotidyltransferase, glycosyltransferase, leucyl-tRNA synthetase, and the NERD protein. Five proteins were found to contain the winged helix-like DNA-binding domain, a nucleic acid-binding motif known to facilitate transcription, helicase activity, and protein–protein interactions. This domain was present in the DNA-directed RNA polymerase of the reference network. Four additional proteins contained the DNA/RNA polymerase superfamily domains, including a Group II intron reverse transcriptase/maturase, a member of a family involved in DNA replication, repair, and retrotransposition. These domains are evolutionarily conserved across polymerases such as DNA Polymerase I, reverse transcriptase, and RNA-dependent RNA polymerases.

We also identified three sequences containing the ribonuclease H-like domain, found in transposase proteins (IS3, IS4, IS231S families), which plays a key role in removing RNA primers during replication and in RNA-DNA hybrid cleavage. Domains corresponding to proteins that provide the necessary substrates and resources such as glycosyltransferase 2-like, nucleotidyltransferase, and leucine–tRNA ligase proteins were also identified to be components of the DNA processing module.

Importantly, proteins identified through our analysis exhibited lineage-specific variation. The NERD protein sequence WP_000453399.1 was exclusive to B clade plasmids, whereas WP_000453400.1 and WP_002069126.1 were found in A and C clades. Similarly, lineage specificity was observed for the Group II intron reverse transcriptase/maturase protein, with WP_001099012.1 occurring only in B clade plasmids and WP_042511945.1 and WP_001099011.1 restricted to A and C clades. Given their known roles in DNA processing, these lineage-associated variants may reflect adaptive phenotypic traits, though further functional validation is required.

Collectively, these results suggest that the identified proteins form a conserved DNA processing module on the pXO1 plasmid. While not exhaustive of all DNA-interacting proteins, this domain-based analysis provides a biologically grounded framework for exploring plasmid replication mechanisms and their phylogenetic implications in *B. anthracis*.

## 4. Discussion

This study demonstrates the insights gained from applying machine learning to investigate the evolutionary and functional dynamics of proteins encoded along the *B. anthracis* pXO1 plasmid. Our multi-scale analysis spanning phylogenetic reconstruction, protein composition, machine learning-based classification, and domain-level annotation underscores the potential of plasmid-based analysis to understand *B. anthracis* function, population structure and evolution. Phylogenetic trees constructed from pXO1 plasmid sequences exhibited notable topological inconsistencies compared to those derived from WGS, particularly within A clade sublineages. Although the broad delineation between major ancestral clades (A, B, and C) was conserved, discordant placement of specific sublineages highlighted the challenges of using plasmid sequences as a standalone proxy for pathogenic and geographic ancestry. Phylogenetic incongruencies likely reflect distinct evolutionary pressures acting on plasmid-encoded virulence factors, as pXO1 carries toxin-encoding genes that are directly involved in host infection and influence transmission dynamics [52,53,54]. The *B. anthracis* lifecycle, characterized by sporulated dormancy and host-dependent replication, creates selective environments where plasmid functions may be subject to localized adaptation pressures distinct from chromosomal evolution [55]. These findings indicate that plasmid evolution is influenced by distinct selective pressures and environments, reinforcing the importance of including spatial epidemiology and pathogenic relevance in analysis with protein language models.

Despite phylogenetic inconsistencies, protein presence–absence vector encodings of the pXO1 plasmid demonstrated clade- and sublineage-specific clustering in a t-SNE projection. Silhouette score analysis confirmed that many sublineages, particularly those with restricted geographic distributions, form compact and coherent clusters. These results indicate that plasmid gene content retains phylogenetic signals that reflect chromosomal evolutionary history.

Association rule mining refined by mutual information analysis identified protein features that effectively distinguish *B. anthracis* sublineages. Consistent with prior work, the key virulence determinants, such as the edema factor and lethal factor emerged as informative features along with the VirB4 protein, a protein involved in plasmid mobilization and conjugative transfer [38,39,56,57]. Beyond identifying known anthrax genes, the module analysis revealed previously unrecognized DNA replication components, including an initiator replication domain–containing protein. This protein, identified through reannotation of hypothetical proteins using embedding-based alignments, is a critical factor in DNA replication and binding in bacteria [58]. These findings underscore the biological relevance captured by our machine learning–based approach and were refined into a decision tree classifier that defines plasmid markers capturing both phylogenetic and functional signals, providing practical PCR-based targets for rapid anthrax identification in settings where whole-genome sequencing is not feasible.

The protein modules defined by the features in the decision tree classifier exhibited both lineage-restricted and sublineage-specific compositional patterns consistent with the WGS phylogeny. Module reflection of WGS phylogeny organization demonstrates the potential of utilizing plasmid encoded features for evolutionary differentiation. For example, distinct alleles of replication-relaxation family proteins and UTP-glucose-1-phosphate uridylyltransferases (GalU) were restricted to specific lineages, while the anthrax edema factor differentiated sublineages at several decision points. By linking allelic variants to lineage classification outcomes, the protein modules complement the decision tree framework and provide tractable targets for experimental validation of anthrax lineage differentiation.

Geospatial analysis of protein module occurrence interpreted in light of epidemiological information reveals patterns and provides context to regional pXO1 module conservation and divergence. Previous studies have linked anthropogenic movement to the evolution and global distribution of *B. anthracis* [5], and features identified within our protein modules reinforce these hypotheses. For example, modules specific to the A.Br.Aust.94 sublineage are identical between Europe and North America. The Tsiankovskii sublineage, restricted to China, uniquely carries the VirB4 family type IV secretion system protein (WP_038357429.1), while the A.Br.WNA sublineage, found only in North America, exclusively encodes the anthrax toxin edema factor allele WP_000197747.1. These geographically constrained signatures represent valuable targets for developing hypotheses about localized selective pressures and distinct evolutionary histories.

Replication-related proteins are conserved across geographically distant strains. This trend is evident in the A.Br.008/011 sublineage, which, despite its broad distribution, retains allelic conservation of the replication-relaxation protein variant WP_000837861.1 and initiator replication domain-containing protein WP_000557418.1 across Europe, South America and Asia. Therefore, our modules demonstrate sublineage conservation of replication-related proteins across regions, indicating the relevance of replication proteins to lineage distinctions. Differences in edema factor alleles among African and European Ancient A strains support the hypothesis of divergent evolutionary trajectories within the same sublineage that are distinct from the modules in remaining A-lineage strains that were spread around the globe. Similar patterns were observed in group B strains, where geographically distant samples shared module features reflective of ancestral retention despite physical distance.

Domain-based analysis of recurrent and ambiguously annotated proteins in the protein modules, such as nucleotidyltransferases and helix-turn-helix (HTH) proteins, indicated distinct subfunctions with complementary biological roles. The co-occurrence of nucleotidyltransferase domain-2 with aminoglycoside resistance domains suggests a modular antibiotic resistance strategy encoded on the pXO1 plasmid. This underscores the capacity of our protein modules to identify under-characterized proteins that warrant further investigation to provide insight into their roles in plasmid function.

Domain-guided annotation of DNA-processing proteins uncovered conserved functional modules present in all pXO1 plasmids analyzed. This module included elements of replication, repair, transcription, and RNA metabolism, with several proteins exhibiting lineage-specific variants. These findings underscore the dual nature of pXO1 as both a virulence and replication vector, whose conserved core functions are essential for plasmid maintenance and pathogenesis while also serving as markers of shared and divergent evolutionary forces.

Ultimately, the plasmid-level markers identified through our approach offer potential markers for public health surveillance, particularly in resource-limited settings where whole genome sequencing may not be readily available. These markers can serve as diagnostic tools for rapid strain characterization and epidemiological tracking, providing specific targets for genomic analysis and field-applicable molecular diagnostics.

## 5. Conclusions

This integrative analysis demonstrates that pXO1 plasmid composition captures biologically and phylogenetically informative patterns in *B. anthracis*. The identification of clade- and sublineage-specific modules, supported by domain-level functional annotation and geographic distribution patterns, highlights the co-adaptive evolution of plasmid protein modules. While plasmid data alone may not suffice for the high-resolution evolutionary analysis required, its functional content offers valuable insights into the ecological and pathogenic dynamics of *B. anthracis*. Future work should explore the mechanistic basis of the identified protein modules and expand this framework to additional virulence plasmids across *Bacillus* and other soil-dwelling pathogens. Application of the computational tools developed here to genome-wide module analysis can identify more comprehensive module arrays that are defining the evolution and pathogenesis of organisms like *B. anthracis.*

## Figures and Tables

**Table 1:** Table of proteins, predicted functions and domains from the STRINGDB NERD gene co-occurrence reference network. Colors correspond to nodes in Figure 7.

STRINGDB Reference Proteins	DNA Processing Superfamily/Domain	Domain Function	Found in pXO1 Plasmid Proteins
Nuclease-related domain containing protein	- NERD: IPR011528	- DNA processing, and may have a nuclease function.	- Nuclease-related domain containing protein
Leucine–tRNA Ligase	- Rossmann-like alpha/beta/alpha sandwich fold: IPR014729	- Bind nucleotide cofactors	- Phosphoadenosine phosphosulfate reductase family protein
Glycosyltransferase 2-like domain-containing protein domains	- Nucleotide-diphospho-sugar transferases: IPR029044	- catalyze the transfer of sugar moieties from an activated nucleotide sugar donor to a specific acceptor molecule.	- UTP–glucose-1-phosphate uridylyltransferase GalU
	- Glycosyl transferase family 2: IPR001173		- Hyaluronan synthase HasA
	- TPR-like: IPR011990	- act as scaffolds for protein–protein interactions, regulating diverse biological processes like cell cycle control, gene regulation, and protein transport.	- Rap family tetratricopeptide repeat protein

	- Tetratricopeptide repeats: IPR019734		
Nucleotidyltransferase domain protein	- Nucleotidyltransferase: IPR043519	- catalyze the transfer of a nucleotidyl group from a nucleotide triphosphate (NTP) to an acceptor molecule in DNA and RNA processing, DNA repair, and signal transduction.	- Nucleotidyltransferase domain-containing protein

			- RelA/SpoT domain-containing protein
DNA Polymerase I	- Ribonuclease H-like: IPR012337	- cleaves RNA within RNA-DNA hybrid structures in DNA replication and repair.	- IS3 family transposase
	- Ribonuclease H: IPR036397		- IS4 family transposase
			- IS4-like element IS231S family transposase
	- DNA/RNA polymerases: IPR043502	- This domain is characterized by its “palm” subdomain, which is crucial to the catalytic activity involving nucleic acid synthesis from a template.	- Group II intron reverse transcriptase/maturase
DNA-Directed RNA Polymerase	- Winged helix-like DNA binding domain superfamily: IPR036388	- sequence-specific DNA binding by transcription factors, as strand-separating wedges in DNA recombination and repair helicases, and can also mediate protein–protein interactions	- Anthrax toxin expression trans-acting transcription regulator AtxA
			- Metalloregulator ArsR/SmtB family transcription factor
			- IS3 family transposase
			- Transcriptional repressor PagR
			- DNA translocase FtsK

## Data Availability

All the code and the data used in this analysis are available on GitHub (https://github.com/Hawaii-Bioinformatics/B-anthracis-pXO1-analysis/: accessed on 28 August 2025).
